# Immune Cell Reaction Associated with *Coenurus cerebralis* Infection in Sheep with Particular Reference to ELISA as a Diagnostic Tool

**DOI:** 10.3390/life12101515

**Published:** 2022-09-28

**Authors:** Soliman M. Soliman, Nesreen H. Aljahdali, Kamlah Ali Majrashi, Sohila M. El-Gameel, Elshaimaa Ismael, Heba M. Salem, Mahmoud A. Mahmoud, Najah M. Albaqami, Haifaa A. Mahjoub, Mohamed T. El-Saadony, Marwa M. Attia

**Affiliations:** 1 Department of Medicine and Infectious Diseases, Faculty of Veterinary Medicine, Cairo University, Giza P.O. Box 11221, Egypt; 2 Department of Biological Science, College of Science, King Abdulaziz University, University Avenue, Jeddah 21551, Saudi Arabia; 3 Biological Sciences Department, College of Science & Arts, King Abdulaziz University, Rabigh 21911, Saudi Arabia; 4 Parasitology Department, Faculty of Veterinary Medicine, Cairo University, Giza P.O. Box 11221, Egypt; 5 Department of Veterinary Hygiene and Management, Faculty of Veterinary Medicine, Cairo University, Giza P.O. Box 11221, Egypt; 6 Department of Poultry Diseases, Faculty of Veterinary Medicine, Cairo University, Giza P.O. Box 11221, Egypt; 7 Department of Pathology-Faculty of Veterinary Medicine, Cairo University, Giza P.O. Box 11221, Egypt; 8 Department of Biological Sciences, Zoology, King Abdulaziz University, Jeddah 21589, Saudi Arabia; 9 Department of Agricultural Microbiology, Faculty of Agriculture, Zagazig University, Zagazig 44511, Egypt

**Keywords:** *Coenurus cerebralis*, ELISA, gene expression, mRNA analysis, TNF-α, IFN-γ

## Abstract

**Simple Summary:**

Infected sheep with *Coenurus cerebralis* (*C. cerebralis*) were subjected to histopathological, hematological, immunological and serological examination. In histopathological sections, cerebral tissue showed an areolar cyst wall with many protoscolices attached to the tissue with necrosis and inflammatory cells’ aggregation. The infected sheep exhibited a significant alteration in blood profile when contrasted with apparently healthy sheep. Evaluation of ELISA specificity using two antigens showed specificities of 46.2% and 38.5% for fluid and scolex Ag, respectively. Meanwhile, accuracy ranged from 76.7% and 73.3% for fluid and scolex Ag, respectively. Levels of TNF-α and IFN-γ were significantly elevated in infected sheep when contrasted with control ones.

**Abstract:**

Sturdy is a disease caused by *Coenurus cerebralis* (*C. cerebralis*) that typically affects the brain and spinal cord of sheep. So, this study aimed to detect the pathological, hematological and immunological changes caused by *C. cerebralis* in sheep. On examination, a total of 17 sheep out of 30 sheep (56.7%) from various regions in Egypt were found infected with *C. cerebralis* from May to August 2019. Each cyst was extracted from the sheep brain; in addition, tissue specimens were taken from the brain tissues for histopathological examination. The hematological profile was analyzed. Enzyme-Linked Immunosorbent Assay’s (ELISA) specificity and sensitivity were evaluated using cystic fluid and protoscolices antigens (Ag). The cell-mediated immunity against the *C. cerebralis* cyst was also assessed via quantitative Real Time—Polymerase Chain Reaction (qRT-PCR) to show alterations in mRNA expression of the Tumor Necrosis Factor-alpha (TNF-α) and gamma Interferon (IFN-γ) cytokines qRT-PCR. In histopathological sections, cerebral tissue showed an areolar cyst wall with many protoscolices attached to the tissue. The affected part showed prominent necrosis together with inflammatory cells’ aggregation. Hyperplastic proliferation of the ependymal cells was a common finding. The infected sheep exhibited significantly lower total erythrocyte numbers (ER), hemoglobin levels (Hb), packed cell volume (PCV), platelet numbers (PN) and segmented cell numbers compared to apparently healthy sheep. Despite the sensitivity for the indirect ELISA being 100% for both of the Ags (fluid and scolex), the evaluation of ELISA specificity using the two antigen (Ag) preparations showed specificities of 46.2% and 38.5% for fluid and scolex Ag, respectively. Meanwhile accuracy ranged from 76.7% and 73.3% for the fluid and scolex Ags, respectively, that showed the priority was directed to the fluid to be used as an ideal sample type for ELISA. Levels of TNF-α and IFN-γ were significantly elevated in infected sheep compared to non-infected control ones. In conclusion, *C. cerebralis* is a serious disease infecting sheep in Egypt revealing economic losses. Although this investigation supports preliminary information about the prevalence, pathological and serological characterization of *C. cerebralis*, further sequencing and phylogenetic analysis is needed to understand better the T. multiceps epidemiology in ruminants and canines in Egypt.

## 1. Introduction

Gid (sturdy) is a fatal infection of small ruminants because of *C. cerebralis*; the larval stage of *Taenia multiceps* that infects the small intestines of dogs and wild canids [1]. *C. cerebralis* cysts’–the disease’s key player–predilection niche is the central nervous system (CNS) of small ruminants causing ovine coenurosis that is presented in Africa and Asia, with an incidence of 1.3 to 9.8% [2].

Coenurosis has two forms: acute and chronic. Acute coenurosis occurs during larvae migration in CNS [3], usually near ten days post the ingestion of contaminated food with a huge count of *T. multiceps* eggs. Acute disease is mainly common in lambs aged 6–8 weeks as the symptoms are found with inflammation and allergic expression, including fever, fatigue, head pressing, convulsions and death [4].

Chronic cases suffer from paralysis, blindness, nystagmus, lethargy, circling movement, lateral deviation of the head (head tilting) and head shaking with no reaction to stimuli. Diseased sheep are separated from the flock by pushing their head versus fixed things that is commonly known as head pressing [5,6]. In addition, coenurosis represents a significant economic loss [7,8].

Locally, the overall prevalence rate of coenurosis in the examined sheep population in Egypt varied from 2.3% to 3.7% [5,9]. The variation in disease prevalence depends on the diagnostic tool, either clinically (about 11%) or by postmortem examination (3%) [5]. In Egypt, coenurosis was more prevalent in sheep [10,11,12], with no molecular information. 

*Coenurus cerebralis* induces different pathological lesions in the brain tissue, including perivascular lymphocytes’ aggregation, neuronal degeneration and demyelination, necrosis and giant cells’ formation and ependymal cells’ hyperplasia of the brain ventricles [12,13]. 

Red blood cell counts (RBCs) and PCV are affected by nutritional status, season, age, parasitic infection and physiological stage (Adewuyi and Adu, 1984). In addition, Mbassa and Poulsen [14] recorded that eosinophilia and basophilia in adult sheep have been considered an indicator of allergic reaction to recent parasitism and investigated the effect of parasitic infection of *O. ovis* on the complete blood cells of sheep (CBC).

The diagnostic specificity of serological methods needs further studies, especially in endemic areas with *Fascioliasis* and *Schistosomiasis*, as the cross-reaction of different parasites’ antigens caused a failure to establish a reliable diagnostic method [15]. The base of using ELISA recombinant Ag in diagnosis of coenurus cysts showed low sensitivity [16]. However, serodiagnosis via indirect ELISA was frequently employed in the induced infection of coenurus in sheep [17]. So, assessment and purification of the Ags are required to enhance the efficacy of these serological methods [18]. 

Serological techniques, such as ELISA and Dot-ELISA, could be used as a screening instrument to detect antibodies against *Coenurus* spp. cysts before living animals’ exports and discover the infection for the early treatment of the disease [19], including identifying and eliminating the focal reservoirs of infection during control programs [20]. 

IFN-γ and TNF-α are mostly influencing cell-mediated Th1 immunity. This response is useful in decreasing the parasitism and stimulation of latent infections, as in the case of *Toxoplasma gondii* and *Neospora caninum* illness [21] through cyst formation and *Oestrus ovis* infection [22].

Therefore, this investigation aimed to describe the brain change because of the cyst infection. In addition, (ELISA) sensitivity and specificity was evaluated, using cystic fluid and protoscolices (PSC) Ag in the serodiagnosis of coenurosis infection in naturally infected sheep. In addition to that, the cell-mediated immunity versus coenurosis in sheep was estimated via evaluation of the modification in mRNA expression of the TNF-α and IFN-γ using qRT-PCR.

## 2. Material and Methods

### 2.1. Animals

A total of 50 sheep from different localities in Egypt were examined according to Constable et al., [23] from May to August and animals that showed nervous signs were recorded. The suspected diseased sheep were slaughtered and subjected to PM exanimation [24].

### 2.2. Sampling

The brain samples were obtained after PM examination from sheep with typical nerve signs and those suspected to be clinically diseased. A sagittal cut of every suspected sheep head was established to detect C. cerebralis in their brains. All of the harvested cysts were processed according to Attia et al. [25] and Varcasia et al. [26].

Cysts were extracted from the infected sheep brains and thoroughly rinsed with saline. Cystic fluid was drawn from the cyst and saved until use. Part of the protoscolices extracted from the cysts was saved in phosphate buffer saline (PBS) pH 7.2, while the other part was kept in 10% formalin buffer and loaded on slides for morphometric assessment. 

In parallel, blood samples were recovered from each tested animal into EDTA-treated and plain 5 mL tubes. The recovered blood was centrifuged at 3500 rpm/15 min, and the sera were harvested and kept at −20 °C for further examination. 

Fecal samples were collected from all examined animals for further parasitological examination. All samples (cysts, whole blood, sera, and faeces) were submitted to the Faculty of Veterinary Medicine, Cairo University, for further examinations. All animal handling steps followed the National Guidelines for the care and use of animals.

### 2.3. Parasitological Examination

A thin Giemsa-stained blood smear was performed to detect the existence of any heme-parasitism associated with *Coenurus* spp. infection. Feces were also tested to discover any gastrointestinal parasitism by following the concentration method [27]. Briefly, about 1 gm of faeces was mixed with 4 mL of saturated salt solution in a 20 mL conical glass test tube, then stirred well and more salt solution was added till the container was nearly full, while the stirring was continued. Any coarse matter which floated up was removed and the tube was placed on a levelled surface with a glass slide being placed over the top of the tube, which was in contact with the fluid. It was allowed to stand for 30 min. The slide was removed and observed for the presence of eggs/cysts.

### 2.4. Histopathological Examination

Tissue specimens were collected from the brain tissues of the animals showing nervous signs and parasitic infection with *C. cerebralis*. The tissue was then fixed in formalin buffered solution 10% and processed for histopathological examinations, according to Bancroft and Gamble [28].

### 2.5. Hematological Examination

To study the effect of coenurosis on the blood cells and platelets count, whole blood was used for CBC counts and PCV value detection, as recorded by Polizopoulou [29].

### 2.6. Serological Study (Indirect ELISA)

#### 2.6.1. Antigen Preparation

The fluid from the *C. cerebralis* cyst was collected and centrifuged at 1500 rpm/15 min at 4 °C, with the supernatant kept in aliquots at 20 °C until needed. The protoscolices were rinsed for 3 times in PBS (PH 7.4) before being re-suspended in an equal amount of PBS and kept at −20 °C until needed. The cyst fluid Ag (HCF) was processed using the Maddison et al. [30] technique. Protoscolex Ag was processed according to Ahmed et al. [31].

#### 2.6.2. Indirect ELISA

A checkerboard titration was utilized to determine the lowest Ag dilution [32], with 96 ELISA flat-bottomed plates. All dilutions and chemicals were administered following [33] on ELISA plates. A total of 10 mg of Ortho-Phenylene Diamine (OPD) substrate and Protein A conjugate (IgG) (Sigma -Aldrich, Cat: A9647, St. Louis, MO, USA) were used. Utilizing the stopping buffer 3N H_2_SO_4_, the reaction was stopped. The optical density (OD) was measured using an ELISA reader set to 450. (Bio-Rad, USA). When the OD value was equal to or exceeded the cut-off value, all sera were counted as positive.

### 2.7. Estimation of TNF-α and IFN-γ

The parasitic-infected tissues were dissected under aseptic conditions. As negative controls, samples from five uninfected sheep were gathered in the same way.

#### 2.7.1. RNA Isolation

According to the manufacturer’s guidelines, the RNA kit (Ambion, Applied Biosystems) was used to isolate RNA from 100 mg of infected samples. A Fast Prep-24 homogenizer (MP Biomedicals, 2 cycles of 30 s.) homogenized the samples in Lysing Matrix D tubes (MP Biomedicals). The quality and amount of RNA were estimated by Nanodrop (Thermo Scientific). Following the manufacturer’s instructions, 500 ng of RNA were prepared by DNaseI amplification grade (Invitrogen). The high-capacity cDNA Archive Kit (Applied Biosystems) was utilized to transcribe the treated RNA. 

#### 2.7.2. qRT-PCR Procedures

Following the sequences submitted to the Gene Bank, specialized PCR primer sets for IFN-γ and TNF-α specific for sheep were created (Table 1). A reference gene, β-actin, was employed for sample normalization. The gene expression was assessed on a different pool of cDNA derived from 5 healthy animals screened for parasites. Denaturation, annealing and extension proceeded as follows: 94 °C for 30 s, then 60 °C for 30 s. followed with 72 °C for 45 s in a 40-cycle amplification, respectively.

### 2.8. Statistical Analysis

Hematological and immunological parameters were compared between healthy and infected sheep using one-way analysis of variance (ANOVA), means and standard error (SE). To assess the sensitivity, specificity, accuracy and other diagnostic values of the Ag, the cut-off point for positive ELISA results was adopted as the two-fold mean optical density (OD) of negative control sera [34]. 

It was considered that the post-mortem examination of slaughtered sheep was the most reliable way to detect *Coenurus* infection, so it was used as the standard gold method [35]. 

To compare the degree of agreement between screening tests, the Kappa statistic (*κ*) was used, following Altman [36] that was executed using the Landis and Koch scale [37]. The Landis and Koch scale scores are classified into no agreement, slight, fair, moderate, substantial, almost perfect and perfect agreement as; <0, 0.0–0.20, 0.21–0.40, 0.41–0.60, 0.61–0.80, 0.81–0.99 and 1.0, respectively [37]. 

A statistical correlation was carried out by PASW Statistics, Version 18.0 computer software (SPSS Inc., Chicago, IL, USA). A *p*-value of < 0.05 was statistically significant.

## 3. Results

### 3.1. Parasitological Examination

The collected blood smear and fecal samples from animals infected with *coenurosis* were negative for blood and gastrointestinal parasites. 

### 3.2. Clinical and Postmortem (PM) Findings

**Figure 1:**
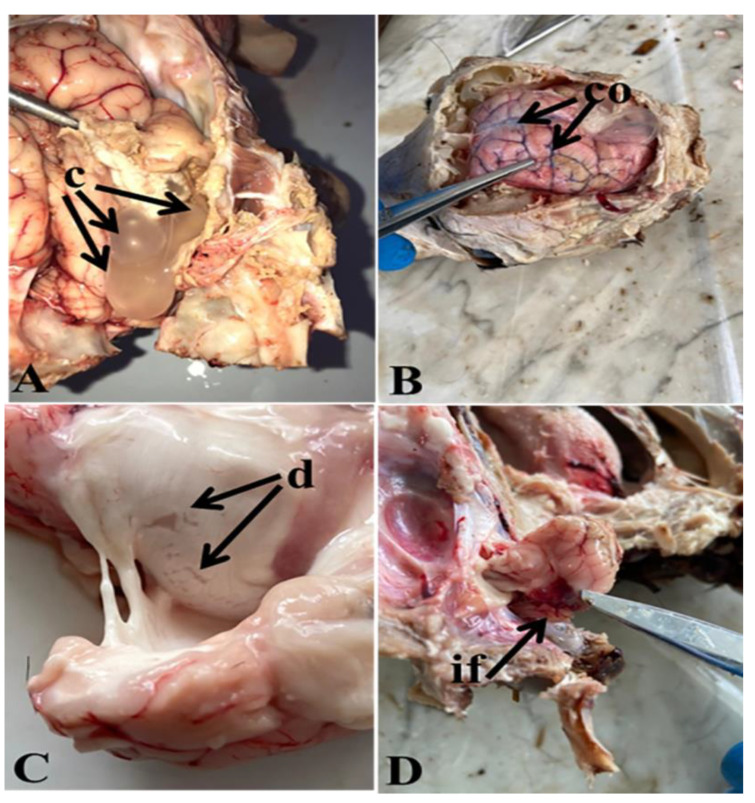
Brain of sheep infected with *C. cerebralis* showing (**A**): Multiple parasite cysts (c) with prominent pressure atrophy of the cerebral tissue. (**B**): Severe congestion (co) of cerebral blood vessels. (**C**): Denuded neuroepithelial (d) surface of the lateral ventricle of the brain. (**D**): Triangular area of hemorrhage on the cerebellum near the site of *C. cerebralis* cyst attachment.

Depending on the fact that clinical prevalence may greatly vary from the postmortem findings, not all of the sheep suffering the clinical nervous manifestations had cysts in their skull. Thirty sheep out of the 50 examined sheep showed variable nervous manifestations such as head tilting, dropping of ears, circling, blindness and head pressing and recumbency. The clinical prevalence of coenurosis was 60.0%. Meanwhile the results of PM examination of the 30 sheep with nervous signs revealed that 17 sheep (56.7%) were positive for *C. cerebralis* infection (cerebral hemispheres and the cerebellum cysts) (Figure 1). 

### 3.3. Postmortem Examination of Collected Cysts

The recovered *C. cerebralis* cysts from infected brains were identified macroscopically as bladder-like and all cysts were single cysts with no evidence of multiple cysts. In addition, cysts were made up from transparent hyaline envelopes with various internal protoscolices immersed in a translucent fluid. The scolices per cyst differed in count from 20 to less than 100 scolices.

### 3.4. Pathological Examination

#### 3.4.1. Gross Pathology

In the brain of sheep infected with *C. cerebralis*, the cerebral tissue showed multiple parasitic cysts with prominent pressure atrophy (Figure 1A) and associated with severe congestion of the cerebral blood vessels (Figure 1B). A pale triangular area of infarction in the cerebrum was also noticed. In the sectioning of the brain, the denuded neuroepithelial surface of the brain’s lateral ventricle was a characteristic finding (Figure 1C). A triangular area of hemorrhage on the cerebellum near the attachment site of the *C. cerebralis* cyst was observed (Figure 1D).

#### 3.4.2. Histopathological Findings

**Figure 2:**
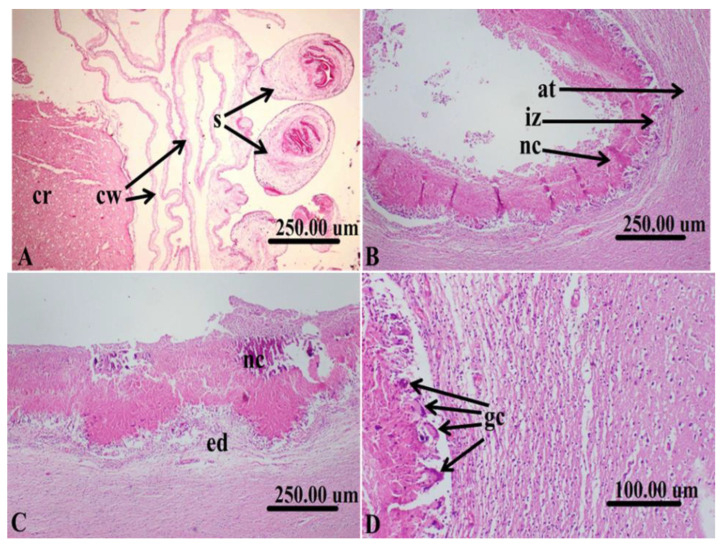
Histopathological sections of cerebrum of sheep affected with *C. cerebralis cyst* showing, (**A**): areolar cyst wall (cw) with many protoscolices (s) attached to the cerebral tissue (cr). (**B**): Area of infarction with prominent necrosis (nc), inflammatory zone (iz) and atrophied tissue. (**C**): Severe necrosis of the tissue (nc) and edema (ed). (**D**): Many multinucleated foreign body giant cells (gc) in the area of inflammatory zone separating the necrotic tissue from the apparently healthy cerebral tissue.

In histopathological sections of the cerebrum of sheep affected with *C. cerebralis* cysts, an areolar cyst wall showed with many protoscolices attached to the cerebral tissue (Figure 2A). The area of infarction with prominent necrosis, inflammatory zones and atrophied tissue were common (Figure 2B). Severe tissue necrosis and edema were observed (Figure 2C). In the area of infarction, many multinucleated foreign body giant cells were noticed in the inflammatory zone separating the necrotic tissue from the healthy cerebral tissue (Figure 2D). 

**Figure 3:**
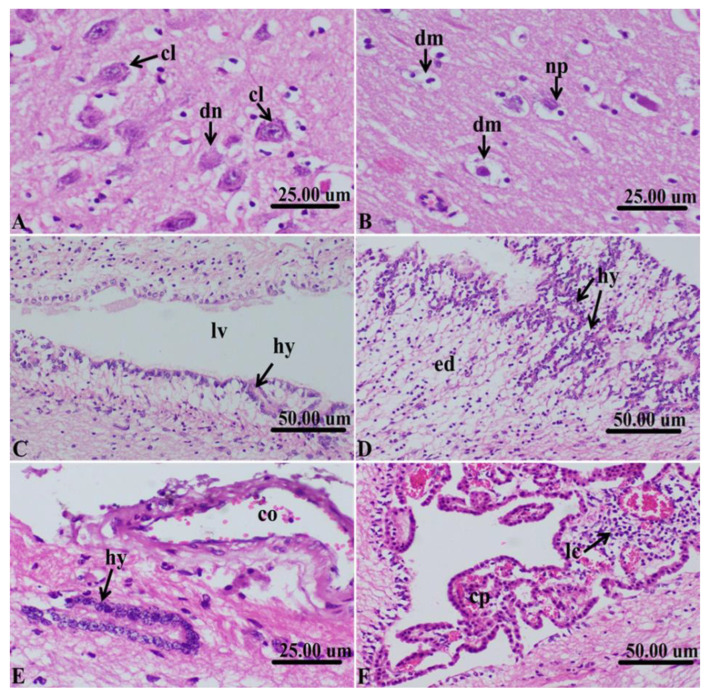
Histopathological sections of cerebrum of sheep affected with *C. cerebralis cyst* showing, (**A**): Chromatolysis of the Nissl’s granules (cl) and dead neurons(dn). (**B**): Demyelinated nerve fibers (dm) and neuronophagia (np). (**C**): Dilation of the lateral ventricle (lv) of the brain and hyperplasia of the neuronal epithelium (hy) and ependymal cells. (**D**): Advanced hyperplasia (hy) of the neuronal epithelium of the lateral ventricle and subepithelial edema. (**E**): Congested newly formed capillaries (co) and hyperplastic cells (hy) invading deep in the brain tissue. (**F**): Severe congestion of the choroid plexus (cp) and perivascular aggregation of inflammatory cells (ic) mainly lymphocytes.

In some other cases, the lesions in the neurons and brain ventricles were the prominent findings, where chromatolysis of the Nissl’s granules and dead neurons were common (Figure 3A) and demyelinated nerve fibers and neuronophagia were observed (Figure 3B). In the brain ventricles, dilation of the brain’s lateral ventricle and hyperplasia of the neuronal epithelium and ependymal cells were noticed (Figure 3C). In other cases, hyperplasia of the neuronal epithelium of the lateral ventricle was advanced and accompanied by subepithelial edema (Figure 3D). In the areas of hyperplasia, congested newly formed capillaries in the hyperplastic tissue were also noticed and the hyperplastic cells invaded deep in the brain tissue (Figure 3E). There was severe congestion of the choroid plexus and perivascular aggregation of inflammatory cells, mainly lymphocytes (Figure 3F). 

### 3.5. Immunological Evaluation of Cell Mediated Immune Response

Infected sheep revealed significantly elevated levels of INF-γ (mean difference = 7 U/mL (29.00 ± 2.08, ±95% C.I.); *t* (4) = 5.269, *p* = 0.006) and TNF-α (8 (±8.96, ±95% C.I.); *t* (4) = 5.422, *p* = 0.006) in contrast with non-infected animals (Table 2).

### 3.6. Hematological Parameters

The hematological findings of examined sheep are found in Table 2. Diseased sheep revealed significantly lower total erythrocyte count, hemoglobin concentration, PCV, platelet count and segmented cell numbers than apparently healthy sheep. Notably, significantly high total leukocytes, eosinophil, staff, lymphocyte and monocyte counts were observed in infected sheep.

### 3.7. Immunological Parameters (Indirect ELISA)

The fluid and scolex Ag cut-off values were 0.37 and 0.41, respectively. Positive optical values for sheep’s sera ranged from 0.53 to 2.30 using fluid Ag, while ranging between 0.50 and 2.90 using scolex Ag. The true prevalence of *C. cerebralis* obtained from PM examination of sheep (Gold standard test) was 17/30 (56.7%). In comparison, 24 (80.0%) and 25 (83.3%) sheep were considered positive for *C. cerebralis* with fluid and scolex Ag, respectively (Table 3). The sensitivity for the indirect ELISA was 100% for both Ag. Furthermore, the specificities were 46.2% and 38.5% for fluid and scolex Ag, respectively. Accuracy ranged from 76.7% to 73.3% for fluid and scolex Ag. 

According to the findings in Table 4, we found that there was a significant moderate correlation between the findings of the indirect-ELISA by fluid Ag and PM examination (*κ* = 0.49, *p* = 0.002), also, between the outcomes of the indirect-ELISA by scolex Ag and PM tests (*κ* = 0.42, *p* = 0.005). There was also a significant, almost perfect agreement between the findings of the indirect-ELISA using the two Ag (*κ* = 0.89, *p* < 0.0001).

## 4. Discussion

Coenurosis is a devasting problem causing major economic losses in the sheep industry. In Egypt, *C. cerebralis* has been described as the main infection responsible for the nervous problems of small ruminants [11] as well as high morbidity and deaths [1,38,39], particularly in young sheep.

The overall prevalence of coenurosis in this study was 56.7% in the examined sheep, *C. cerebralis* (*T. multiceps* metacestode) infection was estimated at 18.3% in the Suez Canal locality [11] and Anwar et al. [10] recorded 100% prevalence in infected sheep from the Cairo governorate, despite negative cysts in the healthy sheep. This is less than the prevalence of 18–100% recorded before in sheep in Egypt [10].

Globally, various occurrences were reported: 44.4% in Tanzania [40], 0.35% in Italy [41] and 18.7% in Iran [42]. The variable prevalence of infection over the various geographical areas might be due to the diversified geographical, ecological and sociological effects [2]. Seasonality is still the most controversial attribute [42,43]. 

The severity of the nervous manifestation of coenurosis might be attributed to the inflammatory response, the affected side of the brain, the space-occupying lesion and the affected centers [44]. In addition, the cyst site and the number of the ingested viable *T. multiceps* eggs are also factors relating to severity [45].

Nervous manifestations from the clinically diseased animals were aligned with those found by [39,46,47,48], who recorded head tilting, corneal opacity usually in one eye, circling, deviated head posture, ataxia and paralysis in some cases.

It is recognized that risk factors affect the prevalence of coenurosis, in particular the locality had a pivotal role as the highest prevalence was monitored in the free-reared areas, as well as the farms that used dogs for protection or those where stray dogs and foxes had access and pastures that were contaminated by the feces of dogs and other carnivores due to the open borders. In contrast, the incidence was low in sheep reared in captivity and kept indoors. 

*C. cerebralis* occurrence is found with the transmission cycle of infected canids. Homeless dogs surround slaughterhouses and those with sheep flocks and wild canids are also likely to contract the infection via access to diseased heads and internal organs cast away during butchering [38,49]. In addition, stray dogs near sheep and goat farms may be fed on dead carcasses on the farms [50]. Unattended non-controlled sheep slaughtering outside slaughterhouses is more prevalent in Egypt, configuring another route of disseminating the infection to dogs. 

*C. cerebralis* is among the serious parasites causing brain pathologies in the small ruminants including sheep [4,12,51]. In our study, the infection with *C. cerebralis* in the examined sheep showed severe pathological lesions ranging from congestion of the blood vessels to neuronal degeneration, necrosis and edema. Our findings agreed with the previously recorded lesions in case of infection with such a parasite [13]. The infarction of the brain tissue could be attributed to the occlusion of the blood vessels because of the surrounding pressure of the growing cysts. To this end, Haridy et al. [12] recorded brain infarction in the case of *C. cerebralis* cysts infecting ewes and hyperplastic proliferation of the neuroepithelial lining of the brain ventricles’ ependymal cells.

The level of PCV was markedly (*p* < 0.05) reduced in coenurosis-infected sheep in contrast with healthy ones. These findings concur with prior recorded elevated erythrocyte oxidation and damage in diseased sheep with *Fasciola hepatica* and *Trypanosoma evansi* [52,53] and hepatic cystic echinococcosis-infected sheep [54].

The early infectious stage of *T. multiceps* in sheep has no apparent clinical symptoms. So, a demonstration of antibody versus coenurus cyst may be useful for early diagnosis of the disease using ELISA [2]. Our investigation revealed that the indirect ELISA’s specificity and accuracy using fluid Ag (46.2%) was higher than using scolex Ag (38.5%). False positive results may be attributed to recent infection, recently developed cysts and cross-reactivity with antibodies from other infections [55]. In addition, infected, infertile or calcified cysts and non-specific conjugate can also play a role in the false positive cases [56]. Furthermore, the sensitivity was 100% for both antigens.

In addition, Huang et al. [15] recorded high sensitivity and specificity of 95% and 96.3%, respectively, for 47 examined sera in contrast with the results of necropsy using indirect ELISA based on recombinant transcriptome of *T. multiceps* (rTmP2) and there was no cross-reaction when P2 protein was used for the diagnosis of *Echinococcus granulosus* positive sera. Moreover, the serodiagnosis of coenurosis in experimentally infected sheep using indirect ELISA was successful [17].

From our findings, the infected sheep revealed significantly elevated levels of INF-γ and TNF-α in contrast with non-infested sheep. This is attributed to stimulation of cell-mediated Th1 immune response versus intracellular pathogens such as *Toxoplasma gondii* and *Neospora caninum*, as it elevates the expression for splenic INF-γ and TNF-α as a method of host protection [57]. As IFN- γ shares in restricting intracellular replication [58] and activating macrophage-mediated processes to destroy intracellular pathogens, especially early in the disease stages [59]. Meanwhile, TNF-α activates phagocytic cells to control the intracellular parasite multiplication and helps get rid of the parasites [58]. 

## 5. Conclusions

*Coenurus cerebralis* infection was detected with a 56.7% prevalence in the sheep under study, in Egypt. In histopathological sections, cerebral tissue showed an areolar cyst wall with many protoscolices attached to the tissue with necrosis and inflammatory cells’ aggregation. The infected sheep exhibited a significant alteration in blood profile when contrasted with apparently healthy sheep. Evaluation of ELISA specificity using two Antigen preparation showed specificities of 46.2% and 38.5% for fluid and scolex Ag, respectively. Meanwhile, the accuracy ranged from 76.7% and 73.3% for fluid and scolex Ag, respectively. Levels of TNF-α and IFN-γ were significantly elevated in infected sheep when contrasted with control ones. Although this investigation supports preliminary information about the prevalence, pathological and serological characterization of *C. cerebralis*, further sequencing and phylogenetic analysis is needed to understand better the *T. multiceps* epidemiology in ruminants and canines in Egypt. 

## Figures and Tables

**Table 1:** The sequences of the forward and reverse primer used in the quantitative real-time PCR.

Gene	Sequence (5′-3′)	Accession Number	Reference
INF-γ	F-CAGAGCCAAATTGTCTCCTTC R-ATCCACCGGAATTTGAATCAG	NM_001009803	Puech et al., 2015
TNFα	F-CCAGAGGGAAGAGCAGTCC R-GGCTACAACGTGGGCTACC	NM_001024860	Puech et al., 2015
β-Actin	F-TGGGCATGGAATCCTG R-GGCGCGATGATCTTGAT	NM_001009784	Puech et al., 2015

**Table 2:** Blood parameters and gene expression analysis in healthy and *C. cerebralis* infected sheep.

Parameters (Normal/Reference Range)	Mean ± SE		*p*-Value
	Control	Infected	
*Blood parameters*			
Total erythrocyte count (9.5–15.0 × 10^6^/c.mm)	11.57 ± 0.88 ^a^	8.23 ± 0.50 ^b^	0.030
Hemoglobin concentration (9–15 g/dl)	10.57 ± 0.09 ^a^	8.50 ± 0.29 ^b^	0.002
Platelet count (800–1100 × 10^3^/c.mm)	850 × 10^3^ ± 28.9 × 10^3 a^	366.7 × 10^3^ ± 66.7 × 10^3 b^	0.003
Packed cell volume (Hematocrit) (27–45%)	28.33 ± 0.88	29.42 ± 1.99	0.645
Total leukocyte count (4000–8000/c.mm)	4933.33 ± 233.33 ^b^	54,680.00 ± 2915.94 ^a^	0.003
Basophils (0–3%)	0.0	0.67 ± 0.67	0.423
Eosinophils (0–10%)	1.67 ± 0.33 ^b^	15.00 ± 1.53 ^a^	0.001
Staff (0%)	0.0 ^b^	4.33 ± 0.88 ^a^	0.039
Segmented (10–50%)	14.00 ± 2.08 ^a^	5.00 ± 0.58 ^b^	0.014
Lymphocytes (40–55%)	45.00 ± 2.89 ^b^	78.33 ± 0.88 ^a^	<0.0001
Monocytes (0–6%)	4.00 ± 0.58 ^b^	17.33 ± 1.45 ^a^	0.001
MCV (28–40 fl)	33.33 ± 2.60	33.33 ± 0.33	1.000
MCH (8–12 pg)	8.53 ± 0.19 ^b^	10.03 ± 0.15 ^a^	0.003
MCHC (31–34 g%)	32.67 ± 0.33 ^a^	30.13 ± 0.19 ^b^	0.003
Gene *expression analysis*			
IFN-γ	5.33 ± 0.88 ^b^	29.00 ± 2.08 ^a^	<0.0001
TNF-α	5.50 ± 0.50 ^b^	23.67 ± 6.51 ^a^	0.009

^a,b^ Different superscripts within the same row indicate significant difference at *p* < 0.05; SE: Standard error; IFN-γ: Gamma interferon; TNF-α: Tumor necrosis factor alpha.

**Table 3:** Evaluation of the diagnostic values of *Coenurus cerebralis* antigens used in indirect ELISA compared to PM examination.

Items	Fluid	Scolex
Sensitivity	100%	100%
Specificity	46.2%	38.5%
Type I error (false positive)	53.9%	61.5%
Type II error (false negative)	0.0	0.0
Apparent prevalence	80.0%	83.3%
PPV	70.8%	68.0%
NPV	100%	100%
Accuracy	76.7%	73.3%
LR+	1.9	1.6
LR−	0.0	0.0

PPV: positive predictive value; NPV: negative predictive value; LR+: Likelihood ratio positive; LR−: Likelihood ratio negative; True prevalence determined by PM examination was 56.7% in sheep.

**Table 4:** The agreement between different antigenic materials and PM examination results of sheep samples.

Diagnostic Test	Kappa Value (*κ*)	Agreement	*p*-Value
Fluid vs. PM	0.49	Moderate	0.002 *
Scolex vs. PM	0.42	Moderate	0.005 *
Fluid vs. Scolex	0.89	Almost perfect	<0.0001 *

* Significant correlation is indicated at *p* < 0.05.

## Data Availability

The data presented in this study are available on request from the corresponding authors.
